# Access Site Related Vascular Complications following Percutaneous Cardiovascular Procedures

**DOI:** 10.3390/jcdd8110136

**Published:** 2021-10-22

**Authors:** Johanna Hetrodt, Christiane Engelbertz, Katrin Gebauer, Jacqueline Stella, Matthias Meyborg, Eva Freisinger, Holger Reinecke, Nasser Malyar

**Affiliations:** Department of Cardiology I―Coronary and Peripheral Vascular Disease, Heart Failure, University Hospital Muenster, Cardiol, 48149 Muenster, Germany; j.hetrodt@icloud.com (J.H.); christianemaria.engelbertz@ukmuenster.de (C.E.); katrin.gebauer@ukmuenster.de (K.G.); jacqueline.stella@ukmuenster.de (J.S.); matthias.meyborg@ukmuenster.de (M.M.); eva.freisinger@ukmuenster.de (E.F.); holger.reinecke@ukmuenster.de (H.R.)

**Keywords:** percutaneous cardiovascular procedures, access site complications, femoral access, pseudoaneurysm, outcome

## Abstract

Vascular access site complications (ASC) are among the most frequent complications of percutaneous cardiovascular procedures (PCP) and are associated with adverse outcome and high resources utilization. In this prospective study, we investigated patients with postprocedural clinical suspicion of ASC evaluated by duplex ultrasound (DUS) for the presence of ASC. We assessed the incidence, in-hospital outcome, treatment of complications and predictors for ASC. Overall, 12,901 patients underwent PCP during a 40 months period. Of those, 2890 (22.4%) patients had postprocedural clinical symptoms of ASC and were evaluated using DUS. An ASC was found in 206 of the DUS examined patients (corresponding to 7.1% of the 2890 DUS examined patients). In 6.7% of all valvular/TAVI procedures, an ASC was documented, while coronary, electrophysiological and peripheral PCP had a comparable and low rate of complications (1.2–1.5%). Pseudoaneurysm (PSA) was the most frequent ASC (67.5%), followed by arteriovenous fistula (13.1%), hematoma (7.8%) and others (11.7%). Of all PSA, 84 (60.4%) were treated surgically, 44 (31.6%) by manual compression and 11 (7.9%) conservatively. Three (0.02%) patients died due to hemorrhagic shock. In conclusion, femoral ASC are rare in the current era of PCP with PSA being the leading type of ASC. Nonetheless, patients with predisposing risk factors and postprocedural suspicious clinical findings should undergo a DUS to early detect and mitigate ASC-associated outcome.

## 1. Introduction

Coronary, electrophysiological, peripheral and valvular diagnostic and interventional percutaneous cardiovascular procedures (PCP) constitute the cornerstone of invasive cardiovascular disease (CVD) management. Even though the radial artery access has proven to be associated with fewer complications [1], femoral access is still widely used, and it is particularly needed for high-risk procedures requiring large-bore sheaths [2,3]. Postprocedural hemostasis of femoral access site is usually achieved either by manual compression or, increasingly, by the use of vascular closure devices. Though rare, percutaneous arteriotomy for PCP can lead to serious access site complications (ASC), the most common of which are pseudoaneurysms (PSA), hematomas, arteriovenous fistulas (AVF), dissections and vascular closure device related local stenoses or occlusions. The consequences include prolonged hospital stay, increased patient morbidity and mortality and higher treatment costs [4].

The aim of this study was to determine the contemporary rate of ACS in symptomatic patients, potential predictors and outcome of puncture-site related vascular complications in patients who underwent diverse types of PCP for diagnosis and therapy of CVD at a tertiary cardiovascular center in Germany.

## 2. Materials and Methods

### 2.1. Patient Selection and Clinical Assessment

We prospectively evaluated all PCP conducted via the femoral access at the University Hospital Muenster, a tertiary cardiovascular center in Germany, from 09/2011 until 12/2014. Patients with symptoms and/or conspicuous clinical examination for an ASC (visible hematoma, swelling or murmur on auscultation at the puncture site) following PCP were screened per duplex-ultrasound (DUS) for detection of ASC such as PSA, AVF, hematoma and others, which include stenosis/occlusion or dissection of the accessed vessels, abscess and complication of closure device. All ASC were managed according to the standard of care as instructed in the standard operating procedures of the institution. If more than one ASC were detected, the most clinical relevant ASC as the leading type was noted.

Information regarding patients’ medical history and previous medication were extracted from the medical records. Procedural data including the vascular access site, the type of procedure and periprocedural administration of medication were assessed from the PCP protocols.

Clinical and procedural factors were investigated for their predictive capabilities for the incidence of all ASC and, particularly, of PSA. The study was approved by the local ethical committee.

### 2.2. Statistical Analysis

Descriptive statistics was applied to all the collected data. Mean and standard deviation were calculated for continuous variables, and the absolute and the relative frequency in percentage for discrete variables. All continuous variables were tested for normal distribution.

In order to identify risk factors for the occurrence of PSA, an explorative logistic regression analysis was performed in which patient characteristics and clinical parameters considered to be particularly relevant were used as predictors. To identify parameters associated with need for surgical treatment of ASC also a logistic regression analysis was performed. A two-tailed *p* value of less than 0.05 was considered significant. All analyses were performed using SPSS 25 software (IBM SPSS Statistics 25, Chicago, IL, USA).

## 3. Results

**Figure 1:**
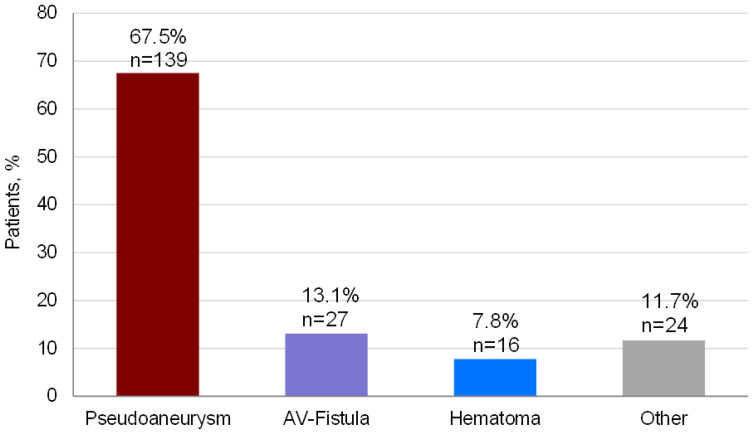
Type and frequency of access site complication. Pseudoaneurysm was the most frequent access site complication with 67.5% of all complications. Arteriovenous (AV) fistula, hematoma and other complications comprised between 7.8% and 13.1% of all access site complications. In patients with more than one access site complication, e.g., an AV fistula and a hematoma, only the most clinical relevant access site complication as the leading type was noted.

During a period of 3 years and 4 months, a total of 12,901 PCP conducted via femoral access were performed, of which 6118 (47.4%) were coronary angiographies/interventions, 3843 (29.8%) electrophysiological testing/ablation, 2474 (19.2%) peripheral vascular angiographies/interventions and 466 (3.6%) valvular procedures, mainly transcatheter aortic valve implantation (TAVI) (Table 1). Due to symptoms and/or conspicuous findings during inspection and auscultation following PCP, 2890 (22.4%) patients were evaluated per DUS for detection of any ASC. In 206 (7.1% of DUS examined patients, corresponding 1.6% of the whole study population) an ASC was found, of which 139 (67.5%) were PSA, 27 (13.1%) AVF, 16 (7.8%) hematoma and 24 (11.7%) other complications (Figure 1).

The frequencies of the different cardiovascular procedures, the number of the corresponding ASC and of the complications for each procedure type are illustrated in Table 1.

The rate of ASC following PCP was highest for valvular/TAVI procedures (6.7%). Coronary, electrophysiological and peripheral PCP had a comparable and low rate of ASC (1.2–1.5%).

For patients with ASC, baseline characteristics and laboratory results at admission are presented in Table 2. Among all patients with ASC, 119 (57.8%) were male, mean age was 71.8 ± 12.7 years and mean body mass index (BMI) was 26.8 ± 5.0 kg/m^2^. Coronary artery (CAD), peripheral artery (PAD) and cerebrovascular (CVD) disease were present in 104 (50.5%), 47 (22.8%) and 14 (6.8%) of all patients, respectively. Renal insufficiency (eGFR < 60 mL/min/1.73 m^2^) was present in 84 (41.8%) patients, and 5 (2.4%) were on dialysis.

### 3.1. Procedural Characteristics of Patients with ASC

Detailed procedural characteristics of patients with ASC are presented in Table 3. Right groin was the access site in most of the cases (*n* = 143; 69.4%) and the common femoral artery as the most frequently used access vessel (*n* = 137; 66.5%), followed by the femoral vein (*n* = 40; 19.4%), the superficial femoral artery (*n* = 22; 10.7%), the deep femoral artery (*n* = 2; 1%) and the common femoral artery and common femoral vein combined (*n* = 2; 1%). The majority of procedures (*n* = 182; 88.3%) were elective.

The procedures had a mean duration of 82 ± 59 min, mean sheath size was 6.9 ± 3.1 FR, mean fluoroscopy time was 873 ± 77 s, with a mean radiation dose of 4248 ± 4657 Gy and a contrast medium amount of 108 ± 82 mL. Vascular closure devices were used in 117 (57.9%) of ASC cases, in 4 cases information concerning use of a closure device was missing. Angio-Seal^®^ was the most frequent closure device type (*n* = 46; 39.3%).

### 3.2. Type of Complication

The type and frequencies of the ASC are illustrated in Figure 1. Among all ASC, PSA was the most frequent complication (*n* = 139; 67.5%), followed by AVF (*n* = 27; 13.1%) and local hematoma (*n* = 16; 7.8%). All other complications such as access-site stenosis/occlusion, local infection, persistent local pain, dysesthesia summed up to 24 (11.7%) cases. During the study period, 3 (0.02%) patients died due to hemorrhagic shock with massive retroperitoneal bleedings, all 3 undetected prior to lethal exit.

### 3.3. Postprocedural Management of Complications

Mean hospital stay of patients with an ASC was 10 ± 9 days. Except for PSA, all other complications such as AV-fistula, hematoma, access-site stenosis, local infection, persistent local pain and dysesthesia were treated conservatively without any intervention. In patients with PSA (*n* = 139), the first manual compression therapy was successful in 22 (15.8%) and a repeat manual compression the next day was successful in additional 22 (15.8%) cases. Of the remaining patients with PSA and at least 2 unsuccessful manual compression attempts, 11 (7.9%) were treated conservatively and surgical intervention was performed in 84 (60.4%) of all cases.

### 3.4. Outcome

**Figure 2:**
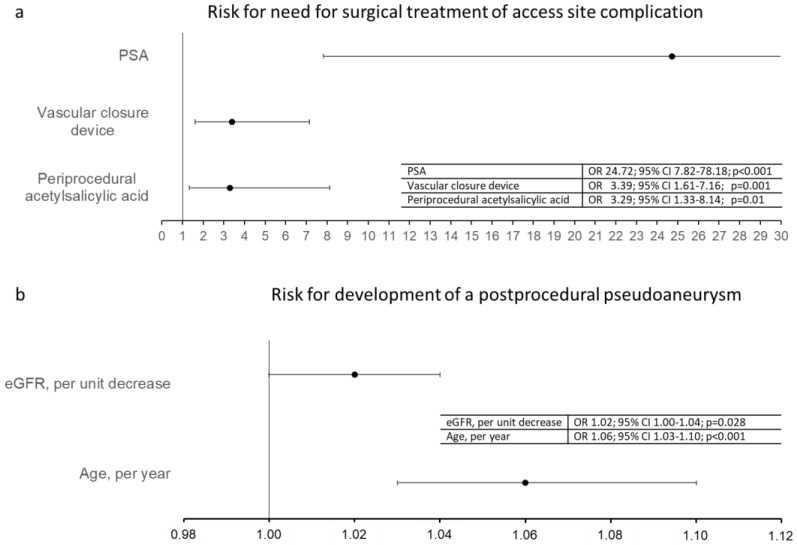
Forest plot showing risk factors for outcome. (**a**): Risk factors for need for surgical treatment of access site complication. Pseudoaneurysm (PSA), vascular closure device and periprocedural acetylsalicylic acid were identified as risk factors by logistic regression analysis. (**b**): Risk factors for the development of a postprocedural pseudoaneurysm Decreasing eGFR and increasing age were identified as risk factors by logistic regression analysis.

There was no sex-related tendency for specific ASC. Factors associated with need for surgical treatment of ASC were PSA (OR 24.72, 95% confidence interval (CI) 7.82–78.18; *p* < 0.001), use of vascular closure devices (OR 3.39, 95% CI 1.61–7.16; *p* = 0.001) and periprocedural use of acetylsalicylic acid (OR 3.29, 95% CI 1.33–8.14; *p* = 0.01, Figure 2a).

Regression analysis showed no significant effect characteristics such as sex, BMI, type of procedure, catheter size (in French), preprocedural international normalized ratio, and perioperative administration of heparin on the occurrence of PSA. Age and renal function (as indicated by the glomerular filtration rate), however, were significantly associated with postprocedural PSA (Figure 2b). Patients with postprocedural PSA were older (age 73.9 ± 10.7 years) than patients with other complications (67.5 ± 15.4 years). Every year increase of life increased the risk for developing a PSA (OR 1.06, 95% CI 1.03–1.10; *p* < 0.001) and each unit decrease in eGFR increased the risk to 1.02 (OR 1.02, 95% CI 1.00–1.04, *p* = 0.028, Figure 2b).

## 4. Discussion

### 4.1. Procedure Types and Complication Rate

Access site complications following PCP are among the most frequent periprocedural complications associated with adverse outcome and increased utilization of resource. In this prospective analysis of femoral ASC following diverse types of PCP at a tertiary cardiovascular center, we observed an overall complication rate of 1.6%, which is lower than previously described [5]. The reported cumulative incidence for ASC in the literature varies, ranging from 0.05 to 21 per 100 interventions [6]. The variation is dependent on the intervention type, the population studied, the used case definition and on the use of diagnostic tools for detection of ASC [7,8,9,10]. In a meta-analysis of 30 studies, the rate of ASC was 1.5% to 9%, of which 20% to 40% required surgical intervention at the access site [7]. Other studies indicated even a higher complication rate of up to 17% [10]. A recent study performed by the University Hospital of Bonn, Germany, reports 1.9% of vascular complications following PCI [11]. Putting our data into perspective, the total PCP-related ASC-rate of 1.6% is lower than compared to the historical data, indicating a time- and experience-dependent decrease of ASC. However, it must be considered that we did not perform a systematic DUS-evaluation of all patients but only in those who had a clinically suspicious finding after the procedure. We might have missed some asymptomatic and clinical unapparent ASC, therefore underestimating the true burden of ASC following PCP. However, when considering the ASC rate in patients with clinical suspicious findings, the rate increased to 7.1%, which is still within the range of previously published studies.

The highest rate of ASC was observed in patients undergoing TAVI. This is not surprising since TAVI patients are older and have more comorbidities including peripheral vascular disease, which are predisposing factors for ASC [5,12]. In the absence of a clear definition of ASC, previously published data about TAVI-associated ASC range between 6% and 36% [13].

Pseudoaneurysm was the most frequent ASC in our study group encompassing 67.5% of all ASC. In contrast to other ASC, most PSA require some kind of intervention, by either manual compression or surgical intervention. Another successful and minimal-invasive therapy is the injection of thrombin into the aneurysm sac. However, at the time of the study, this was not a routine approach at our institution. This explains the high fraction of surgical intervention following un-successful manual compression for the final treatment of PSA (*n* = 84, 60.4%). The use of a closure device and periprocedural application of acetylsalicylic acid increased the risk for a subsequent surgical intervention by 3.39 and 3.29, respectively.

In the literature, various predictors have been described to correlate with the occurrence of a PSA. A review from 2018 [14] found the following parameters to be associated with the incidence of postprocedural PSA: Advanced age, female sex, increased BMI, low platelet cell count, coronary heart disease, diabetes mellitus, arterial hypertension, hemodialysis, cardiovascular intervention, anticoagulative therapy, puncture of the left groin, catheter sizes 7–12F, too high/too low puncture and insufficient manual compression after puncture. In our exploratory study, however, only age and renal failure as indicated by reduced glomerular filtration rate were predictors of postprocedural incidence of PSA. Patients with preexisting renal failure/chronic kidney disease exhibit higher rates of general and specific access site complications. Moreover, the complication rate is related to the degree of renal impairment [15]. The impact of chronic renal insufficiency and outcomes after percutaneous coronary intervention was analyzed in a large-scale US Nationwide Inpatient Sample database (*n* = 3187,404 patients). The authors demonstrated that in-patients undergoing percutaneous coronary intervention, chronic renal insufficiency was a predictor of higher in-hospital mortality, higher periprocedural complications, longer hospital length of stay and higher costs. Though it is evident that patients with renal failure tend to higher rates of thrombotic events, they also are at higher risk for bleeding and hemostatic disorders such as pseudoaneurysm as well. Renal dysfunction/chronic kidney disease are associated with platelet dysfunction and abnormal soluble coagulation factors. However, the exact pathophysiological mechanisms are not fully understood yet.

This study demonstrates that PSA was not only the most common ASC (67.5% of all complications) but also the most severe with a 24.7 times higher risk of vascular surgery intervention in the course compared to other complications.

Vascular closure devices have been shown to be associated with shorter time of hemostasis, rapid ambulation and fewer ASC compared to manual compression alone to achieve hemostasis at the puncture site [16]. However, as indicated by our data, if there is a PSA following the use of a closure device, the need for a surgical intervention is higher than without using a closure device.

### 4.2. Limitations

Only symptomatic and/or conspicuous patients were examined per DUS. It is most probable that we might have missed some asymptomatic patients with ASC and therefore underestimated the true burden of ASC. The data are derived from a single-center, therefore the generalizability is limited. The incidence and particularly the therapeutic management of ASC might be different in other centers, such as thrombin injection for treatment of PSA instead of surgical intervention.

## 5. Conclusions

The contemporary overall ASC rate following percutaneous cardiovascular procedures at a large-volume, tertiary cardiovascular center is with 1.6% at an acceptable low rate and as shown by historical data has decreased over the years probably due to extended experience of the treating physicians. Particularly patients with suspicious clinical findings after the procedures should be evaluated per DUS to confirm or exclude ASC. Pseudoaneurysms constitute two-third of all ASC and require frequently therapeutic interventions.

## Figures and Tables

**Table 1:** Percutaneous cardiovascular procedures and corresponding access site complication according to procedure type.

	PCP *n* = 12,901	Clinical Suspicion of ASC *n* = 2890	Confirmed ASC *n* = 206
Coronary intervention, *n* (%)	6118 (47.4)	1277 (44.2)	92 (1.5% of all coronary interventions)
Peripheral intervention, *n* (%)	2474 (19.2)	532 (18.4)	37 (1.5% of all peripheral interventions)
Electrophysiological intervention, *n* (%)	3843 (29.8)	645 (22.3)	46 (1.2% of all electrophysiological interventions)
TAVI/ valvular intervention, *n* (%)	466 (3.6)	436 (15.1)	31 (6.7% of all TAVI/valvular interventions)

ASC denotes access site complication; PCP: percutaneous cardiovascular procedures; TAVI, transcatheter aortic valve implantation.

**Table 2:** Baseline characteristics of patients with access site complications proven by duplex ultrasound.

Characteristics	Total (*n* = 206)
Age in years, mean ± SD	71.8 ± 12.7
BMI in kg/m^2^, mean ± SD	26.8 ± 5.0
Male, *n* (%)	119 (57.8)
**Cardiovascular risk Factors and Comorbidities**	
Diabetes mellitus, *n* (%)	44 (21.4)
Current smoker, *n* (%)	29 (14.1)
Previous smoker, *n* (%)	28 (13.6)
Dyslipidemia, *n* (%)	71 (34.5)
Hypertension, *n* (%)	139 (67.5)
CAD, *n* (%)	104 (50.5)
PAD, *n* (%)	47 (22.8)
CVD, *n* (%)	14 (6.8)
Renal insufficiency ^1^, *n* (%)	84 (41.8)
Renal replacement therapy, (%)	5 (2.4)
**Previous Medication**	
Acetylsalicylic acid 100 mg, *n* (%)	70 (34.0)
Clopidogrel, *n* (%)	136 (66.0)
Vitamin-K-Anatgonists, *n* (%)	57 (27.7)
**Laboratory Results**	
Creatinine in mg/dL, mean ± SD	1.27 ± 1.16
Glomerular filtration rate in mL/min/1.73 m^2^, mean ± SD	64.8 ± 24.3
Platelets in thousands/mL, mean ± SD	216 ± 66
International Normalized Ratio, mean ± SD	1.47 ± 0.69
Hemoglobin in g/dL, mean ± SD	12.9 ± 1.9

^1^ Renal insufficiency was defined as the current GFR < 60 mL/min/1.73 m^2^. Information about renal insufficiency and renal replacement therapy was available in 201 patients. BMI denotes body mass index; CAD: coronary artery disease; CHD: coronary heart disease; CVD: cerebrovascular disease; PAD: peripheral artery disease; SD: standard deviation.

**Table 3:** Procedural characteristics of patients with access site complications.

Procedural Characteristics	Total (*n* = 206)
Puncture site	
Left, *n* (%)	63 (30.6)
Right, *n* (%)	143 (69.4)
Punctured vessel	
Common femoral artery, *n* (%)	137 (66.5)
Common femoral vein, *n* (%)	40 (19.4)
Superficial femoral artery, *n* (%)	22 (10.7)
Deep femoral artery, *n* (%)	2 (1.0)
Indication *n* (%)	
Elective, *n* (%)	182 (88.3)
Urgent, *n* (%)	24 (11.7)
Sheath size in French, mean ± SD	6.9 ± 3.1
Vascular closure devices ^1^, *n* (%)	117 (57.9)
Angio-Seal^®^, *n* (%)	46 (39.3) ^#^
FemoSeal^TM^, *n* (%)	22 (18.8) ^#^
StarClose^TM^, *n* (%)	17 (14.5) ^#^
Exoseal^®^, *n* (%)	15 (12.8) ^#^
Prostar^TM^, *n* (%)	15 (12.8) ^#^
Examination duration in min, mean ± SD	82 ± 59
Fluoroscopy time in seconds, mean ± SD	873 ± 77
Radiation dose in Gy, mean ± SD	4248 ± 4657
Contrast medium in mL, mean ± SD	108 ± 82
Hospital length of stay in days, mean ± SD	10 ± 9
Hemoglobin in g/dL, mean ± SD	12.9 ± 1.9

^1^ Information about type of vascular closure device was missing in two cases. ^#^ Percentage refers to 117 patients with vascular closure devices. SD denotes standard deviation.

## Data Availability

Data are stored at an electronic repository at the University Hospital Muenster.
